# Surface Modification of Graphene Nanoplatelets by Organic Acids and Ultrasonic Radiation for Enhance Uremic Toxins Adsorption

**DOI:** 10.3390/ma12050715

**Published:** 2019-03-01

**Authors:** M. Andrade-Guel, C. Cabello-Alvarado, V. J. Cruz-Delgado, P. Bartolo-Perez, P. A. De León-Martínez, A. Sáenz-Galindo, G. Cadenas-Pliego, C. A. Ávila-Orta

**Affiliations:** 1 Centro de Investigación en Química Aplicada, Departamento de Materiales Avanzados, Saltillo 25294, Mexico; marlene.andrade@ciqa.edu.mx (M.A.-G.); gregorio.cadenas@ciqa.edu.mx (G.C.-P.); 2 CONACYT-Consorcio de Investigación Científica, Tecnológica y de Innovación del Estado de Tlaxcala, Tlaxcala 90000, Mexico; christian.cabello@ciqa.edu.mx; 3 CONACYT-Unidad de Materiales, Centro de Investigación Científica de Yucatán, A.C., Mérida 97205, Mexico; victor.cruz@cicy.mx; 4 Centro de investigación y de Estudios Avanzados del IPN-Unidad Mérida, Departamento de Física Aplicada, Mérida 97310, Mexico; pascual.bartolo@gmail.com; 5 Universidad Autónoma de Coahuila, Facultad de Ciencias Químicas, Departamento de Química Orgánica, Saltillo 25280, Mexico; p.leon@uadec.edu.mx (P.A.D.L.-M.); aidesaenz@uadec.edu.mx (A.S.-G.)

**Keywords:** ultrasonic radiation, graphene nanoplatelets, adsorption, uremic toxins

## Abstract

Ultrasound energy is a green and economically viable alternative to conventional techniques for surface modification of materials. The main benefits of this technique are the decrease of processing time and the amount of energy used. In this work, graphene nanoplatelets were treated with organic acids under ultrasonic radiation of 350 W at different times (30 and 60 min) aiming to modify their surface with functional acid groups and to improve the adsorption of uremic toxins. The modified graphene nanoplatelets were characterized by Fourier transform infrared spectroscopy (FT–IR), thermogravimetric analysis (TGA), and X-ray photoelectron spectroscopy (XPS). The optimum time for modification with organic acids was 30 min. The modified nanoplatelets were tested as adsorbent material for uremic toxins using the equilibrium isotherms where the adsorption isotherm of urea was adjusted for the Langmuir model. From the solution, 75% of uremic toxins were removed and absorbed by the modified nanoplatelets.

## 1. Introduction 

Nowadays, Diabetes mellitus, systemic arterial hypertension, increased weight, and obesity have a high impact on human health. All of these can cause chronic kidney disease (CKD), which is a complex disease. CKD represents 70% of all deaths in the US and México, with CKD being one of the main ten mortality causes in the last decade [1]. CKD can be treated by hemodialysis, whose main goal is to supersede the kidney function, which consists in passing blood through a system comprised of a filtration mechanism, where the residuals or by products of the biochemistry and physiology of cellular metabolism are separated [2]. Hemodialysis treatment involves four hours, three days a week, however, several side effects such as headaches and dizziness, among others are also shown. Besides, it is an expensive treatment. A solution to improve the hemodialysis process and give a better quality of life for patients is the development of new adsorbent materials that can be easily incorporated into a polymeric membrane. Zeolite [3] and activated carbon [4], among others, have been used as adsorbent materials for uremic toxins. Multiwalled carbon nanotubes modified with arginine have been used with good results in hemocompatibility and antimicrobial activity, and can be considered candidates for hemodialysis membranes [5]. Other carbon materials are graphene nanoplatelets that have physical and chemical properties such as high surface area, high mechanical rigidity, and high electrical conductivity, among others, allowing their application in different areas [6]. Graphene modification, specifically the modification using ultrasound, helps to deagglomerate nanoparticles and could promote modification [7]. Modified graphene is considered a good adsorbent of arsenic [8], pollutant organic molecules [9,10], and heavy metals [11,12,13], therefore representing an alternative for the removal of uremic toxins. Graphene can be modified with citric acid, however, only 3% of modification is achieved in the case of carbon nanotubes. The percentage of modification can be enhanced using a combination of organic acids from renewable resources, being an alternative to the chemical modification of graphene, since the traditional oxidizing agents like inorganic acids are harmful to the environment. On the other hand, sonochemistry is a technology that has multiple effects such as emulsification, dispersion, activation, and degradation. All these are attributed to the cavitation phenomenon, which is characterized by the formation, creation, and collapse of micro-bubbles during the whole process [14]. The conventional method for graphene modification is the Hummers method, which involves two steps where graphite oxidizes, mainly due to a combination of oxidizing agents (KMnO_4_ or KClO_3_) and intercalation agents (H_2_SO_4_, HNO_3_ or H_3_PO_4_). This process occurs at temperatures between 40–90 °C for 2 h [15]. The advantages of using ultrasonic irradiation in chemical modification are the reduction of reaction times and the increase in the formation of functional groups in the surface of carbonaceous materials [16]. 

The aim of this study is to perform a surface modification of graphene nanoplatelets by a combination of organic acids, citric acid with oxalic acid and citric acid with formic acid, and ultrasonic irradiation with different sonication times to determine the adsorption of uremic toxins on these graphene nanoplatelets. 

## 2. Materials and Methods 

### 2.1. Materials

Graphene nanoplatelets (10 to 12 layers) with a purity of 97%, with a diameter of 2 to 3 µm, identified as industrial-grade graphene nanoplatelets, were purchased at Cheap Tubes, Inc. (Cambridgeport, VT, USA). Citric acid, oxalic acid, and formic acid have a purity of 99% and were acquired from Sigma Aldrich (Toluca, México). Distilled water with a pH of 7 was used in all aqueous solutions.

### 2.2. Methods

#### Chemical Modification of Graphene 

The treatment was performed by dispersing 2 g of graphene nanoplatelets into 100 mL of distilled water with a combination of citric and oxalic acid; or citric and formic acid; in a 1:1 ratio, using a digital ultrasonic processor from Cole-Parmer model CPX750 (Cole-Parmer, Vernon Hills, USA), operated at 20 ± 0.1 kHz, with an output power of 750 W, at an amplitude of 50%, in continuous mode and plugged into a catenoidal titanium horn of 25 mm in diameter. For safety reasons, all experiments were done into a sound-abating enclosure. Two different treatment times of 30 and 60 min were applied. All experiments were performed at room temperature. At the end of the experiments, the graphene nanoplatelets were washed several times with distilled water until it reached a neutral pH, filtered and dried at 80 °C for 24 h.

### 2.3. Characterization Techniques

The materials were characterized by TA Instruments thermogravimetric analyzer (TGA) (TA Instruments, New Castle, USA), which was used to examine the thermal stability of samples within a temperature range from 30 to 600 °C, at 10 °C/min, under nitrogen flow of 50 mL/min. From 600 to 800 °C, the nitrogen flow was changed to oxygen flow, at the same 50 mL/min, in order to obtain the total oxidation of the sample. 

Fourier-transform infrared spectroscopy was recorded using a Magna Nicolet 550 spectrometer (GMI, Minnesota, USA) with 100 scanner and resolution of 16 cm^−1^, in the range of 400 to 4000 cm^−1^. Previously, the samples were dried in a vacuum oven (Perkin Elmer, San Diego, USA) at 100 °C for 15 h, and thereafter they were supported in KBr pellets. The XPS (X-ray photoelectron spectroscopy) study was realized in the K-ALPHA spectrophotometer (ThermoFisher, Massachusetts, USA) (Thermo Scientific, model XL-30 Phillips instrument with an accelerating voltage of 5–25 keV) with a monochromatic X-ray source with binding energy of 0–1350 eV and a depth of 400 μm, there is no pre-treatment to the samples. The X-ray powder diffraction data were collected on a Rigaku-smartlab diffractometer, operating at 40 kV and 40 mA with stability of 0.01%/8 h. Measurements of each system were performed in the scattering 2Θ range of 5° to 70°with a step of 0.02 and counting rate of 10 s/step. The quantification of acid groups was determined quantitatively by its acid-base titration. The methodology for the titration was based on the method of Bohem, which consists of placing 5 mL of a solution of 0.01 N of NaOH in 0.01 N NaCl in a matrix containing 10 mg of oxidized graphene. The samples were sealed and placed on a shaking rack at room temperature for 24 h, and then filtered and titered with 0.01 N HCl in NaCl solution. Table 1 shows the identifications of the samples used in each of the experiments. 

### 2.4. Adsorption of Uremic toxins

The urea and uric acid were dissolved in distilled water separately at different concentrations (20, 40, 60, 80, 100, 120, 140, 160 mg/L) to create a calibration curve, which was read in the UV-Vis spectrometer Shimadzu model UV-1800 (Shimadzu, Duisburg, Germany). 

The adsorption experiments were performed in precipitation glasses of 50 mL with 20 mL of solution of 160 mg/L (urea or uric acid) and 50 mg of unmodified and modified graphene nanoplatelets. The precipitation glasses were placed in a stirring plate at 37 °C and a stirring speed of 100 rpm, for 4 h, similar to the hemodialysis treatment time. Each 15 min a sample was taken and read in the UV-Vis spectrophotometer. The experiments were performed twice for reproducibility; the data given in the manuscript are the average values. Absorbance was measured by sampling at regular intervals. The concentration of uremic toxins in the solution was determined using the Beer–Lambert law by monitoring absorbance versus wavelength with λmax 200 and 293 nm for urea and uric acid

The removal percentage was calculated according to the following equation:

Equation (1).

(1) $$\%\;\mathrm{Removal}=\frac{\left(Ci-Ce\right)}{Ci}\times 100$$

where Ci is the initial concentration and Ce is the final concentration. The adsorption capacity of graphene was calculated with the following equilibrium equation: 

Equation (2).

(2) $$qe=\frac{\left(Ci-Ce\right)V}{m}$$

where V is volume in L liters solution and *m* is the mass in mg of adsorbent.

### 2.5. Adsorption Isotherm

Langmuir and Freundlich models can describe the adsorption equilibrium. To test both models, absorption isotherms data were fitted and the correlation coefficient (R^2^) was calculated using the trendline command in Microsoft Excel. The Langmuir isotherm was calculated using the following equation:

Equation (3).

(3) $$\frac{Ce}{qe}=\frac{Ce}{qm}+\frac{1}{K_{L}q_{m}}$$

where qe (mg g^−1^) and Ce (mg L^−1^) are the concentrations of the solid phase and liquid phase of the adsorbate in equilibrium, respectively, qm is the maximum adsorption capacity and K_L_ is the constant obtained from plotting Ce/qe versus Ce.

The Freundlich isotherm was calculated using the following equation:

Equation (4).

(4) $$\ln q_{e}=\ln K_{F}+\left(\frac{1}{n}\right)lnCe$$

where K_F_ (mg g^−1^) (L mg^−1^) and 1/n are the Freundlich constants related to adsorption capacity and n is the heterogeneity calculated with the lineal plot of lnqe versus lnCe. 

## 3. Results and Discussion

### 3.1. Fourier Transform Infrared Spectroscopy (FTIR)

**Figure 1:**
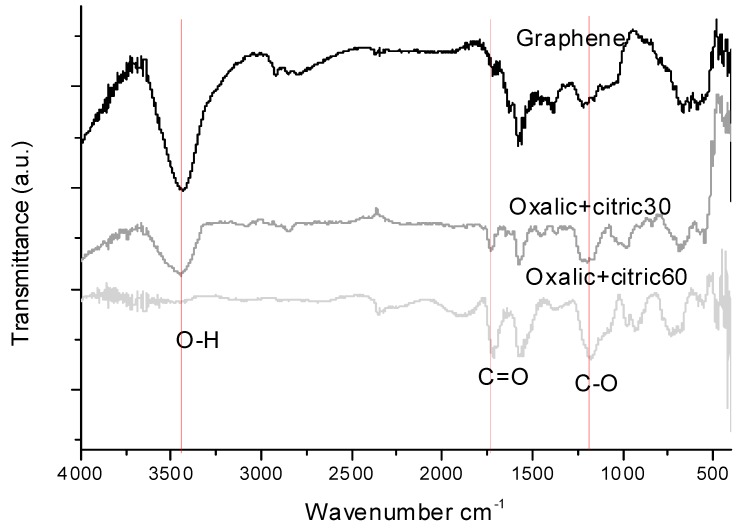
Fourier transform infrared (FT–IR) spectra of the unmodified and modified graphene with Oxalic+Citric30 and Oxalic+Citric60.

Figure 1 shows the FT–IR spectra of the unmodified and modified graphene with oxalic+citric30 and oxalic+citric60. For samples of graphene and oxalic+citric30, one peak at 3430 cm^−1^ corresponding to the bond formed by O-H in the samples was observed. This peak is not observed in the sample oxalic+citric60, likely due to the degradation of graphene and/or acid evaporation induced by the increase in the ultrasonic dose [17]. 

All the samples exhibit the following characteristic signals: C=O stretching (from -COOH) at 1715 cm^−1^ and C-O vibration (from C-OH) at 1178 cm^−1^. These results are similar to those reported by Wang et al 2016 [18] for tartaric acid modified graphene oxide. The adsorption band outstanding at 1715 cm^−1^ of oxalic+citric30 and oxalic+citric60 is due to COOH bond stretching vibration of the carboxylic acid. The FT–IR band at 1575 cm^−1^ was attributed to the carboxylate groups. These characteristic peaks indicate that large amounts of oxygen-containing functional groups exist on the graphene surface. 

**Figure 2:**
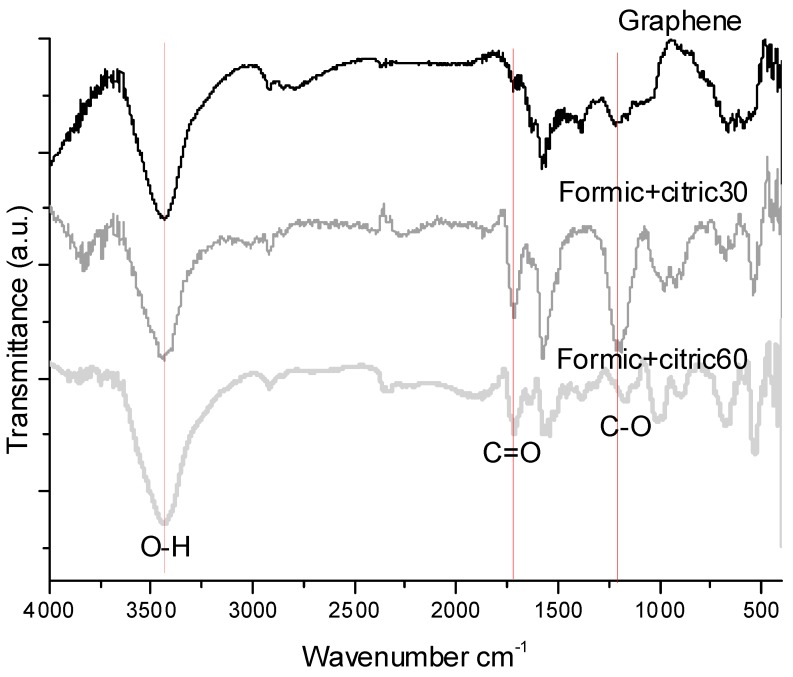
FT–IR spectra of the unmodified graphene and modified graphene with Formic+Citric30, Formic+Citric60.

Figure 2 shows the FT–IR spectra of the unmodified and modified graphene with formic+citric30 and formic+citric60. All samples exhibit the presence of two main bands around 1715 cm^−1^ corresponding to the bond C=O and C-O vibration at 1200 cm^−1^, these bands can be observed in the Formic+citric30 and formic+citric60 samples [19]. Hydroxyl (O-H) groups have an intensive absorption around 3420 cm^−1^, due to the presence of hydroxyl groups [20], as observed for those modified by oxalic + citric acid. The formation of these peaks after the treatment confirm the attachment of acidic functional groups onto the graphene surface. It can be concluded that the optimal time of ultrasonic radiation was 30 min since the adsorption band at 1573 cm^−1^ had a peak that was of greater intensity for the sample formic+citric30 compared to formic+citric60.

### 3.2. X-ray Photoelectron Spectroscopy (XPS)

**Figure 3:**
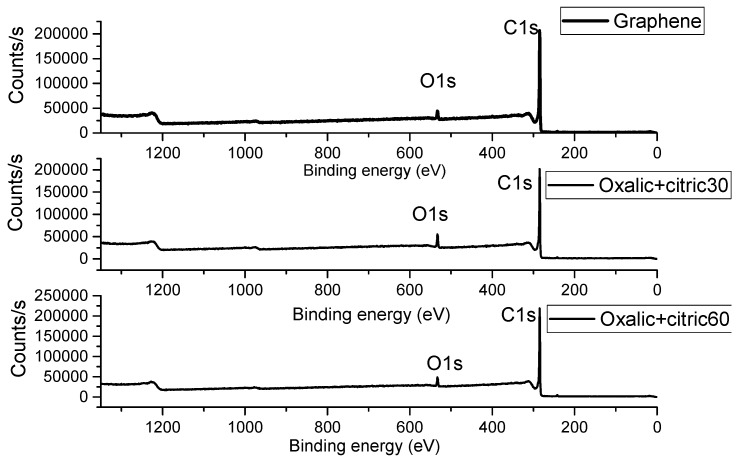
XPS spectrum of unmodified graphene and modified graphene with Oxalic+Citric30 and Oxalic+Citric60.

The XPS spectra of unmodified and modified graphene with oxalic+citric samples are seen in Figure 3. Is noted that in oxalic+citric30 sample possess an oxygen percentage of 8.08%, almost double compared to the value of 4.72% in the unmodified graphene. The C1s signals are present at 285.03 eV for oxalic+citric30 sample and 284.47 eV for oxalic+citric60, as presented in Table 2. This coincides with Kim et al. 2014 [21], who reported the graphene functionalization with phosphonic acid. 

**Figure 4:**
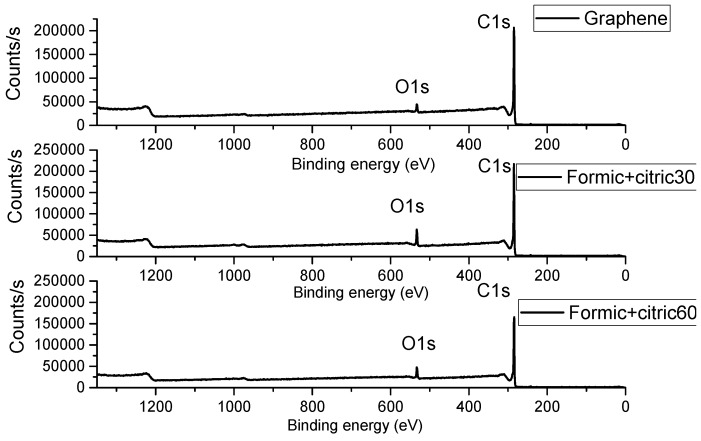
XPS spectrum of unmodified graphene and modified graphene with Formic+citric30 and Formic+citric60.

Figure 4 shows the XPS spectra of the unmodified and modified graphene with formic+citric30, formic+citric60 samples. In the case of the unmodified graphene, the spectrum shows a signal that corresponds to C1s at 285.32 eV and a small signal of O1s at 532.2 eV. These results coincide with the report by Geng et al. 2009 [22] for unmodified graphene. In the modified graphene with formic+citric30 and formic+citric60, there are two signals C1s in 284.94 eV, 284.93 eV and O1s in 532.89 eV, 532.89 eV, respectively, as shown in Table 2. The oxygen percentage increased in both samples and the intensity of the C-C peak due to the sp^2^ carbon bond in graphene gradually decreased due to the addition of carboxylic groups on their surface, thus the chemical state of graphene was modified [23]. The same effect was noted by infrared spectroscopy in which the characteristic signals of the carboxylic group were observed.

**Figure 5:**
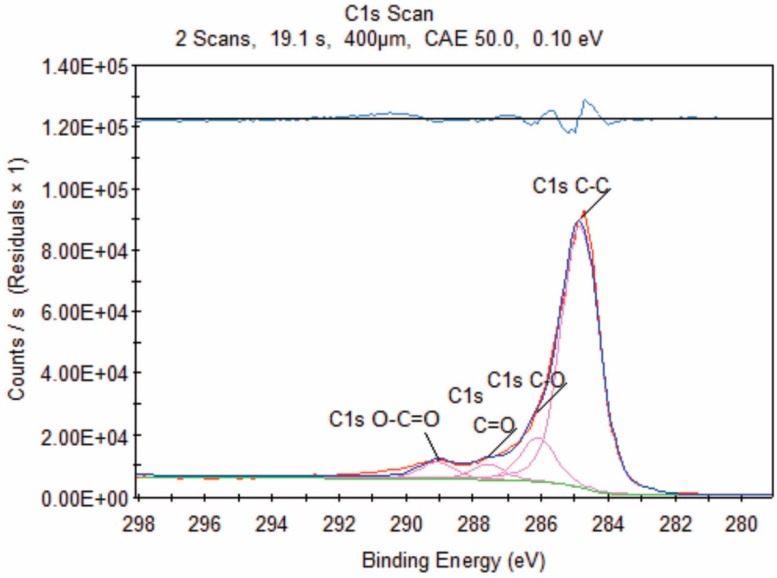
Peak deconvolution of C1s of the graphene modified with oxalic+citric30.

Figure 5 shows the deconvolution of the C1s peak. The main peak has a binding energy of 285.0 eV, which is attributed to C-C, C=C and C-H corresponding to the graphenic structure [24]. The deconvolution has three peaks located at binding energies of 286.4 eV, 287.6 eV, 289.1 eV assigned to C-OH, C=O, O-C-OH species, respectively [25]. The intensity of C-O was greater than C=O and O-C-OH, which can be associated with a larger amount of C-O groups. The hydroxyl and carboxyl groups lead to the formation of the epoxide groups but in the spectrum of XPS is not observed because the oxidation of graphene is lower. The reason for this lower oxidation is that the conventional Hummers method uses KMnO_4_ in H_2_SO_4_ as an oxidizing agent. 

### 3.3. Thermogravimetric Analysis (TGA)

**Figure 6:**
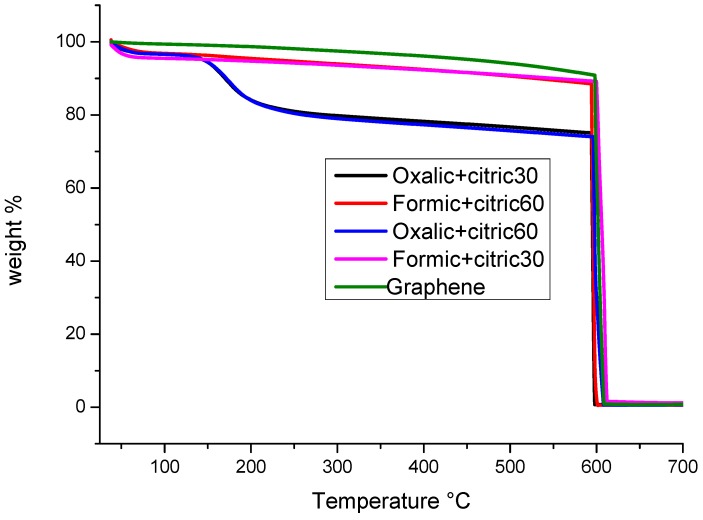
Thermogravimetric analysis (TGA) analysis of graphene and the modified samples with organic acids.

Thermogravimetric analysis is shown in Figure 6. Unmodified graphene shows only a single weight loss at 600 °C and thermal stability up to 1000 °C under non-oxidative atmosphere. This was previously reported by Shang et al. 2012 [26], and they attribute it to carbon pyrolysis. A weight loss of 3% is detected in modified graphene with formic+citric samples from ambient temperature to 75 °C, attributed to the desorption of water molecules on the graphene surface. A second weight loss of about 8% was noted at a temperature ranging from 75 °C to 600 °C, corresponding to desorption of small molecules from the modification process or even some functional groups weak attachment in the nanoplatelets surface. In contrast, for samples of modified graphene with oxalic+citric there were detected three weight losses, the first weight loss from ambient temperature up to 140 °C with 5% in weight attributed to elimination of water molecules in graphene surface, which coincides with Dave et al. 2015 [27]. These authors reported a similar weight loss attributed to functional oxygen groups. The second weight loss occurs from 140 °C to 200 °C, which corresponds to desorption of existing organic groups weakly attached to the graphene surface, with a loss of 15%. Finally, a third weight loss event occurs at 600 °C where graphene degrades into CO_2_ and other compounds.

**Figure 7:**
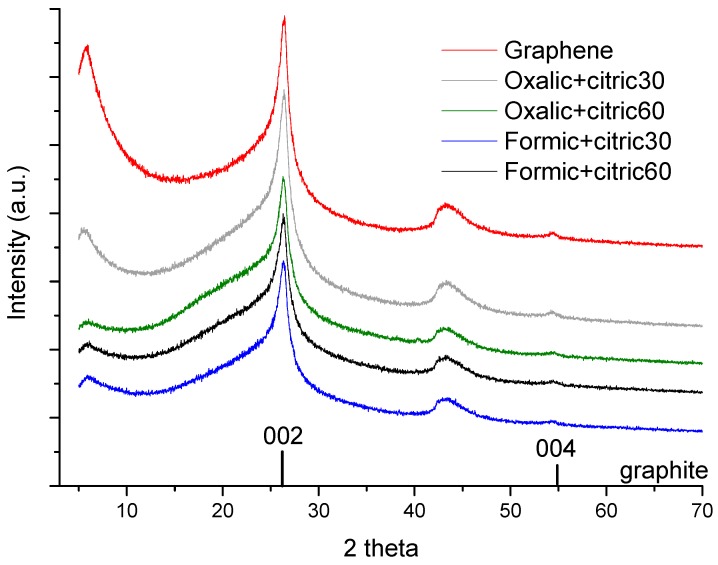
X-ray diffraction patterns of unmodified graphene and modified graphene.

The XRD spectra measured in a range of 2theta from 5° to 70° (Figure 7). The intensity of (002) the peak increases from formic+citric 60 to oxalic+citric30, indicating a higher number of graphitic layers [28]. The patterns showed a larger interlayer spacing of graphene oxide than graphite powder layers due to the insertion of oxygen containing functional groups between the layers [29]. The comparison of the XRD patterns of unmodified graphene and modified graphene showed that the characteristic peak of graphite in the (002) plane associated to 2Ɵ = 26.4° but the modified graphene showed a slightly larger diffraction angle; for example oxalic+citric60 2Ɵ =26.28°, indicating a short range order in the stacked graphene layers [30].

### 3.4. Scheme of Surface Modification of Graphene Nanoplatelets

**Figure 8:**
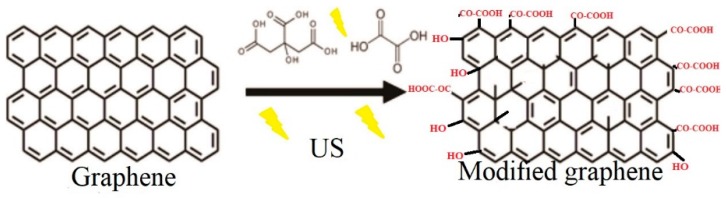
Scheme for the graphene modification under the action of organic acids and ultrasonic radiation.

Based on the TGA, FT–IR, and XPS results, Figure 8 shows a possible scheme for surface change of graphene nanoplatelets, as a result of the modification with a mixture of organic acids (citric and oxalic) and ultrasonic radiation. Different types of defects are present on the graphene surface such as point defects, cluster defects, and boundaries or edges. It is possible that the oxidation of the graphene carbon atoms is taking place due to the oxidizing organic acids producing carboxylic acids and hydroxyl groups on the graphene surface due to its reactivity. Ultrasonic irradiation disperses graphene nanoplatelets and this allows interaction with the reaction medium. Through the acid–base titration it was possible to quantitatively detect the percentage amount of carboxylic groups present in the structure of graphene, while for the unmodified graphene they were not present. The percentages obtained were: Formic+citric30 3.1%, formic+citric60 3.3%, oxalic+citric30 4.7% and oxalic+citric60 4.4%, which agree with XPS results.

### 3.5. Adsorption Study of Uremic Toxins 

**Figure 9:**
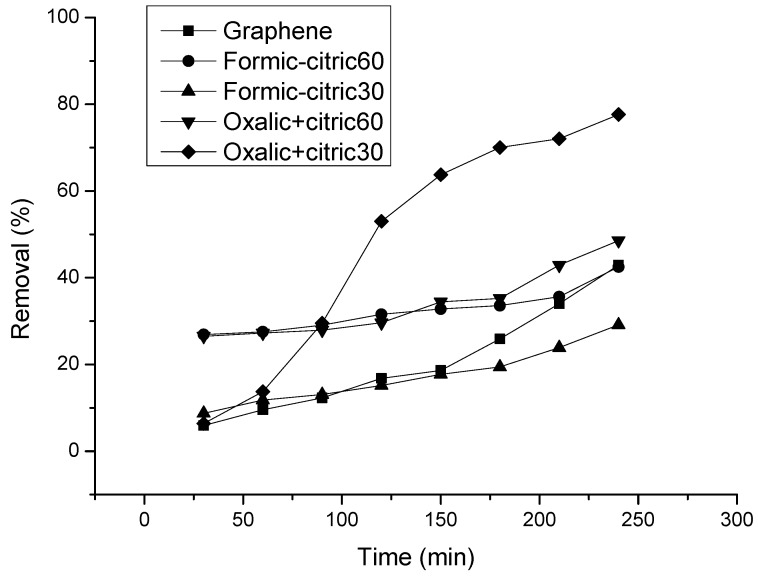
Percentage removal of urea using graphene and modified graphene with organic acids and ultrasonic radiation.

Uremic toxins can be classified based on their principal physicochemical characteristics that affect the solute elimination by dialysis. Special attention has been applied to uremic toxins of low molecular weight like urea and uric acid, which in general are used for adsorption tests [31]. Figure 9 shows the removal percentage of urea in a solution at 4 h of time, the typical hemodialysis treatment time. Is noted that modified graphene with oxalic+citric30 has a removal percentage of 75% while unmodified graphene has just 15% of urea removal. Thus, when surface is modified with acidic sites, the adsorption of urea molecules in the graphene surface is enhanced. Modified graphene with formic+citric at different times, shows removal percentages between 15 and 30%. In order to consider effective any adsorbent material, urea reduction must be at least 60% [32]. In this study, a 75% removal is reported, which converted modified graphene with oxalic+citric30 into a good candidate for urea removal. 

**Figure 10:**
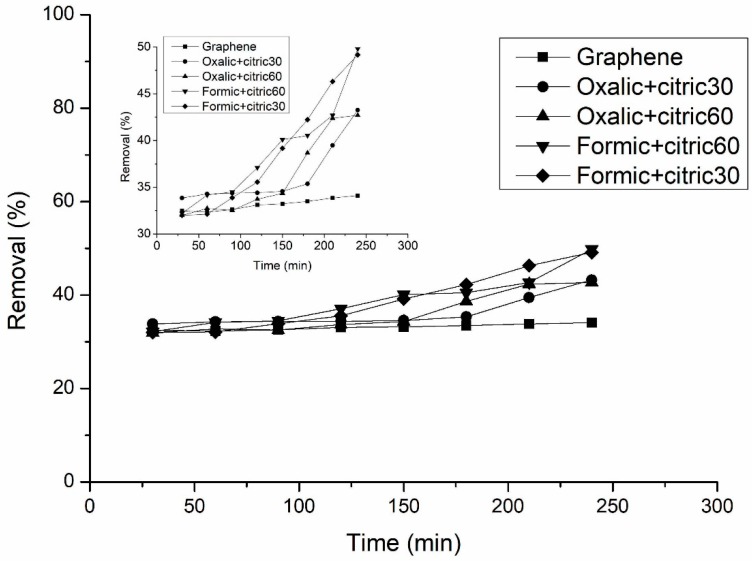
Percentage removal of uric acid using graphene and modified graphene with organic acids and ultrasonic radiation.

Figure 10 shows the percentage removal of uric acid from graphene and modified graphene with organic acids, all samples have values ranging from 30 to 50% of removal. Uric acid is a complex structure which has tautomerism, which hinders their adsorption in different adsorbent materials. Uric acid presents different structures depending on the solution in which it is in, for example, in a basic solution, the molecular form of 2,6,8-trihydroxypurine and negative purine-oxygen ions are predominant; and in a neutral solution, it is possible that all the three forms, uric acid, 2,6,8-trihydroxypurine, and negative purine-oxygen ions, exist simultaneously. In this study, uric acid is present in a neutral solution [33]. Other factors that affect the adsorption of uric acid in materials are temperature and concentration.

**Figure 11:**
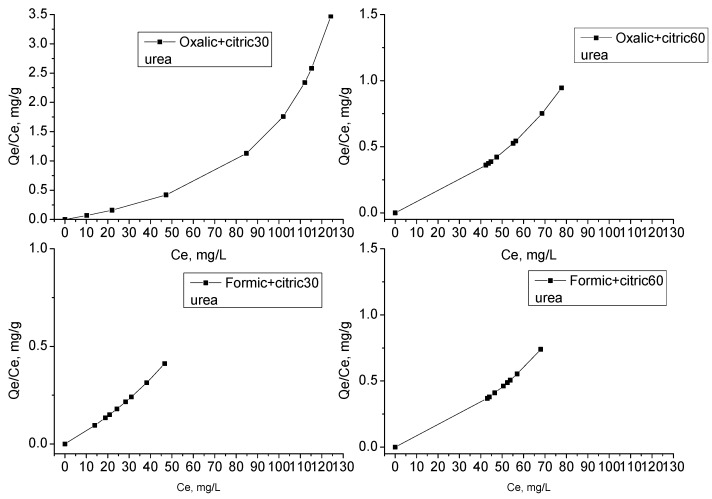
Langmuir model of adsorption for urea on graphene and modified graphene with organic acids samples.

**Figure 12:**
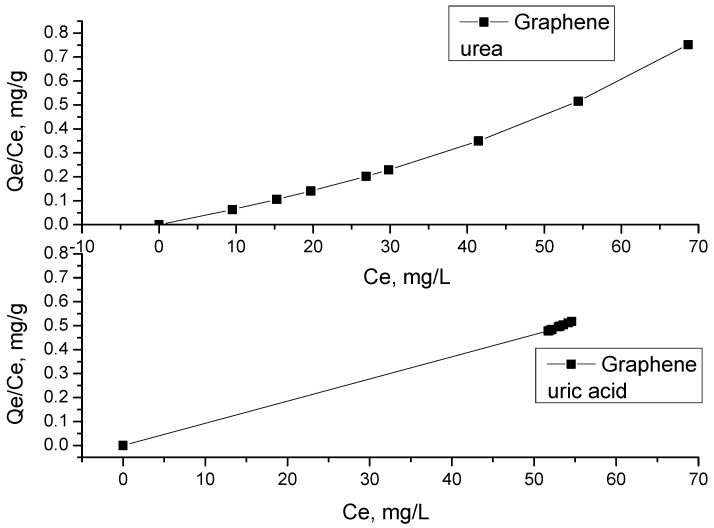
Langmuir model of adsorption for urea and uric acid of graphene.

The result of the adsorption tests was based on the comparison of R^2^. Table 3 shows the possible monolayer adsorption and that this behavior corresponds to a Langmuir isotherm (Figure 11 and Figure 12). The quantity of urea molecules increases until reaching a limit value corresponding to a coating of the surface by a monolayer and is characteristic of a chemisorption process. Instead, for modified graphene formic + citric60 and oxalic+citric60, represents the typical multilayer behavior of a Freundlich isotherm. Gao et al. 2006 [34] reported the synthesis of silica nanocomposites for urea adsorption, which also showed monolayer adsorption.

Table 4 shows the results of uric acid adsorption on unmodified graphene and graphene modified with organic acids. All samples show a typical Freundlich multilayer adsorption, which is carried out with the physical adsorption of uric acid molecules in different layers. There is no limit layer, which is consistent with the adsorption study performed by Liu et al. 2008 [35], where the activated carbon surface was modified with ammonia.

### 3.6. Scheme of Adsorption of Urea on Graphene Nanoplatelets

**Figure 13:**
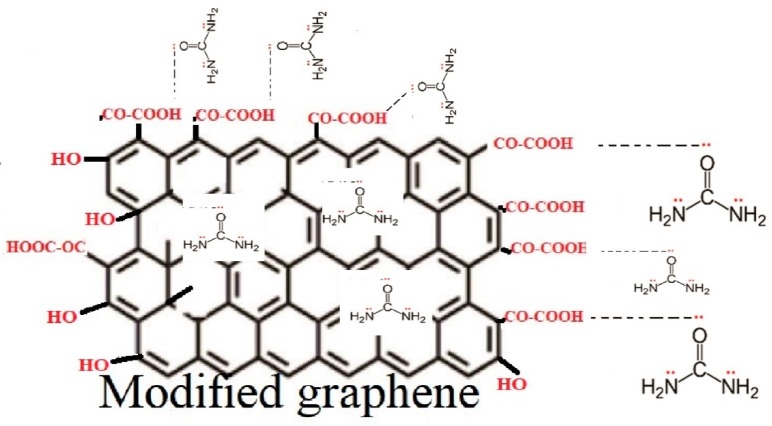
Proposal scheme for urea adsorption onto the modified graphene nanoplatelets.

Based on the above results, the following scheme is suggested for the adsorption mechanism. The acidic functional groups attached to graphene nanoplatelets can bind toxins (urea) by an attraction between the free electron pair of oxygen in the carbonyl functional group with the free electron pair of nitrogen of a urea molecule. At the same time, the hydroxyl functionality of the graphene surface could interact with the amine and carbonyl group of urea (Figure 13) by means of bridging hydrogen bonds. The isotherm of Langmuir proposes a chemical adsorption on the surface of graphene, forming a monolayer. The scheme shows only three molecules on the graphene surface, but it can be coated with these molecules forming a monolayer.

## 4. Conclusions

The modification of graphene with organic acids and ultrasonic radiation was carried out successfully, as revealed by XPS, after deconvolution of the C1s peak, C-O and O-C=O bonds were found. The optimum experimental condition for reaching this modification was a combination of oxalic and citric acids in a ratio 1:1, and a time treatment of 30 min. The ultrasonic radiation promotes the attachment of acidic functionalities on the surface of graphene, but when the exposure time is increased, structural damage could occur.

The advantage of this method compared to that of H_2_SO_4_/HNO_3_ mixtures is the shorter reaction time, as it has been reported that times longer than 1 h for acidic oxidation with H_2_SO_4_/HNO_3_ produces serious modifications of the graphene network, limiting further potential applications. However, this method is considered green because it fulfills two principles of green chemistry, which are the use of renewable raw materials and alternate energies such as ultrasound.

Urea adsorption was achieved in all materials of modified graphene. Oxalic+citric30 material had a high level of adsorption (75%) and adjusted very well with the Langmuir isotherm, indicating that adsorption phenomena occurs by a monolayer formation, and that it is a good candidate as an adsorbent material for hemodialysis treatment. In contrast, uric acid adsorption in all modified graphene samples showed a poor adsorption value of 35%.

The feasibility of adding modified graphene (oxalic and citric acid) to a polymeric membrane for enhancing its efficiency in the adsorption of uremic toxins makes this material an alternative for improving hemodialysis treatment.

## Figures and Tables

**Table 1:** Identification of the samples according to the treatment time.

Sample	Time (min)	Organic Acids Employed	Ratio
Graphene	0	None	0
Oxalic+Citric30	30	Oxalic and Citric	1:1
Oxalic+Citric60	60	Oxalic and Citric	1:1
Formic+Citric30	30	Formic and Citric	1:1
Formic+Citric60	60	Formic and Citric	1:1

**Table 2:** Characteristic signals of photoemitted electrons and their respective atomic percentages.

Sample	C1s Peak (eV)	O1s Peak (eV)	C1s At %	O1s At %
Graphene	285.32	532.89	95.28	4.72
Formic+Citric30	284.94	532.89	93.12	6.88
Formic+Citric60	284.93	532.89	95.02	4.98
Oxalic+Citric30	285.03	533	91.92	8.08
Oxalic+Citric60	284.47	532.81	92.7	7.3

^At %^ Atomic percent; ^eV^ Electronvolts

**Table 3:** Parameters of isotherms for urea adsorption, according with the Langmuir and Freundlich approach.

Sample	Langmuir			Freundlich		
	k	q_max_	R^2^	n	K_f_	R^2^
Graphene	0.010	0.056	0.973	1.050	1.189	0.6769
Oxalic + Citric30	0.024	0.353	0.8892	0.594	6.61	0.3972
Oxalic + Citric60	0.011	0.093	0.9382	1.128	6.68	0.9466
Formic + Citric30	0.008	0.024	0.9858	1.324	6.74	0.8687
Formic + Citric60	0.010	0.056	0.9627	1.168	6.78	0.9769

**Table 4:** Parameters of isotherm for uric acid adsorption, according with the Langmuir and Freundlich approach.

Sample	Langmuir			Freundlich		
	k	q_max_	R^2^	n	K_f_	R^2^
Graphene	0.009	0.007	0.9993	1.17	0.001	0.9996
Oxalic + Citric30	0.010	0.022	0.9752	1.13	0.023	0.9912
Oxalic + Citric60	0.010	0.033	0.9682	1.12	0.042	0.9842
Formic + Citric30	0.011	0.061	0.9444	1.09	0.060	0.9761
Formic + Citric60	0.011	0.073	0.9445	1.09	0.081	0.968
